# CARD9 Deficiency Increases Hippocampal Injury Following Acute Neurotropic Picornavirus Infection but Does Not Affect Pathogen Elimination

**DOI:** 10.3390/ijms22136982

**Published:** 2021-06-29

**Authors:** Suvarin Pavasutthipaisit, Melanie Stoff, Tim Ebbecke, Malgorzata Ciurkiewicz, Sabine Mayer-Lambertz, Theresa Störk, Kevin D. Pavelko, Bernd Lepenies, Andreas Beineke

**Affiliations:** 1 Department of Pathology, University of Veterinary Medicine Hannover, 30559 Hannover, Germany; suvarin.pavasutthipaisit@tiho-hannover.de (S.P.); melanie.stoff@tiho-hannover.de (M.S.); malgorzata.ciurkiewicz@tiho-hannover.de (M.C.); theresa.stoerk@tiho-hannover.de (T.S.); 2 Center for Systems Neuroscience, 30559 Hannover, Germany; tim.markus.ebbecke@tiho-hannover.de (T.E.); bernd.lepenies@tiho-hannover.de (B.L.); 3 Department of Pathology, Faculty of Veterinary Medicine, Mahanakorn University of Technology, Bangkok 10530, Thailand; 4 Institute for Immunology and Research Center for Emerging Infections and Zoonoses, University of Veterinary Medicine Hannover, 30559 Hannover, Germany; sabine-mayer1@gmx.de; 5 Department of Immunology, Mayo Clinic, Rochester, MN 55905, USA; pavelko.kevin@mayo.edu

**Keywords:** Theiler’s murine encephalomyelitis virus, neurotropic virus, CARD9, hippocampal damage

## Abstract

Neurotropic viruses target the brain and contribute to neurologic diseases. Caspase recruitment domain containing family member 9 (CARD9) controls protective immunity in a variety of infectious disorders. To investigate the effect of CARD9 in neurotropic virus infection, CARD9^−/−^ and corresponding C57BL/6 wild-type control mice were infected with Theiler’s murine encephalomyelitis virus (TMEV). Brain tissue was analyzed by histology, immunohistochemistry and molecular analyses, and spleens by flow cytometry. To determine the impact of CARD9 deficiency on T cell responses in vitro, antigen presentation assays were utilized. Genetic ablation of CARD9 enhanced early pro-inflammatory cytokine responses and accelerated infiltration of T and B cells in the brain, together with a transient increase in TMEV-infected cells in the hippocampus. CARD9^−/−^ mice showed an increased loss of neuronal nuclear protein^+^ mature neurons and doublecortin^+^ neuronal precursor cells and an increase in β-amyloid precursor protein^+^ damaged axons in the hippocampus. No effect of CARD9 deficiency was found on the initiation of CD8^+^ T cell responses by flow cytometry and co-culture experiments using virus-exposed dendritic cells or microglia-enriched glial cell mixtures, respectively. The present study indicates that CARD9 is dispensable for the initiation of early antiviral responses and TMEV elimination but may contribute to the modulation of neuroinflammation, thereby reducing hippocampal injury following neurotropic virus infection.

## 1. Introduction

Neurotropic viruses cause infections of the central nervous system (CNS) in humans and animals worldwide that can either be asymptomatic and self-limiting or fulminant and fatal [1,2]. Hippocampal alterations following viral encephalitis may also lead to long-term sequelae, including learning disabilities and an increased risk of developing epilepsy [3,4,5,6,7,8]. Theiler’s murine encephalomyelitis virus (TMEV) is a neurotropic picornavirus that transiently infects the hippocampus, causing neuronal damage in C57BL/6 mice [9,10,11], making TMEV infection a valuable model to study virus-induced neurologic disorders [2,10,11,12,13,14,15]. Virus elimination in C57BL/6 mice is initiated by innate immune responses that contribute to antiviral CD8-mediated antiviral immunity [16,17,18]. However, early innate responses may also promote neuropathology by releasing neurotoxic factors. Thus, early innate responses are a “double-edged sword” and need to be tightly controlled during the course of TMEV infection [13,19,20,21,22,23].

Caspase recruitment domain containing family member 9 (CARD9) is an adaptor protein that delivers signals through immunoreceptor tyrosine-based activation motif (ITAM)-coupled or hemITAM-bearing C-type lectin receptors (CLRs), including Dectin-1, Dectin-2 and Mincle [24,25,26,27]. The Syk/CARD9 signaling axis triggered by engagement of Dectin-1, Dectin-2, or Mincle was recently shown to contribute to early innate responses against certain viruses, but also against bacteria, parasites and fungal pathogens [28,29,30,31,32,33,34]. Viruses, such as vesicular stomatitis virus (VSV), encephalomyocarditis virus and vaccinia virus, which are sensed by the cytosolic nucleic sensors RIG-I, MDA5 and RAD50, respectively, were shown to engage CARD9 for inflammatory cytokine production [35,36,37]. However, in contrast to the predominant protective effect observed in fungal diseases, CARD9 signaling seems to play an ambivalent role in viral disorders, with beneficial effects by promoting antiviral responses (e.g., VSV, vaccinia virus) and detrimental effects by triggering virus-induced immunopathology (e.g., influenza virus, coxsackievirus) being described in animal experiments [36,37,38,39]. So far, the role of CARD9 signaling in the development of brain lesions following neurotropic virus infection has not been determined.

Since several CLRs use the adapter protein CARD9 for intracellular signaling, the aim of this study was to determine the effect of the CLR/Syk/CARD9 axis on virus control and neuropathology. To investigate the impact of CARD9 on acute neuropathology, the TMEV infection model was employed using CARD9^−/−^ mice and C57BL/6 wild-type control mice (WT). Results indicate that CARD9 signaling has a limited role in early antiviral immunity but contributes to reducing hippocampal injury following acute neurotropic virus infection.

## 2. Results

### 2.1. Loss of CARD9 Promotes Hippocampal Damage Following Virus Infection

**Figure 1:**
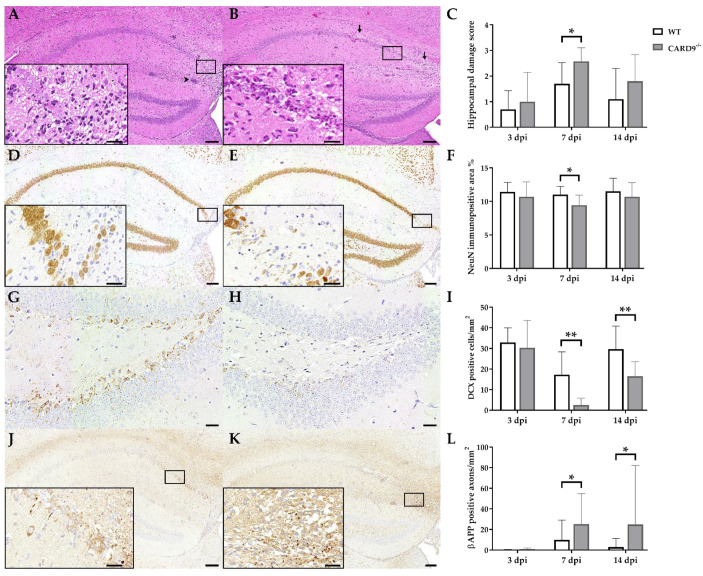
Hippocampal damage in C57BL/6 wild-type (WT) mice and CARD9-deficient (CARD9^−/−^) mice following Theiler’s murine encephalomyelitis virus (TMEV) infection. Hematoxylin and eosin staining of the hippocampus with (**A**) mild neuronal loss and perivascular cuffs (arrowhead) of a WT mouse and (**B**) marked neuronal loss (arrows) of a CARD9^−/−^ mouse at 7 days post infection (dpi). (**C**) Histological scoring of hippocampal damage. NeuN-specific immunohistochemistry of (**D**) a WT mouse with mild loss of neuronal nuclear antigen (NeuN)^+^ mature neurons in the hippocampus and of (**E**) a CARD9^−/−^ mouse with prominent loss of NeuN^+^ neurons in the CA1 region at 7 dpi. (**F**) Percentage of NeuN immunopositive area in the hippocampus. Doublecortin (DCX)-specific immunohistochemistry in the hippocampal dentate gyrus of (**G**) a WT mouse and (**H**) a CARD9^−/−^ mouse with marked loss of DCX^+^ cells at 7 dpi. (**I**) Quantification of DCX^+^ neuronal progenitor cells in the dentate gyrus. β-APP-specific immunohistochemistry of (**J**) a WT mouse with a marginal number of β-APP^+^ axons in the hippocampus and (**K**) a CARD9^−/−^ mice with several swollen β-APP^+^ axons in the hippocampus at 7 dpi. (**L**) Quantification of β-APP^+^ axons in the hippocampus. (**C**,**F**,**I**,**L**) Significant differences between WT and CARD9^−/−^ mice are labelled with asterisks (* *p* ≤ 0.05; ** *p* ≤ 0.01; Mann–Whitney *U*-test). Three dpi: WT *n* = 10, CARD9^−/−^  *n* = 10; 7 dpi: WT *n* = 10, CARD9^−/−^  *n* = 9; 14 dpi: WT *n* = 10, CARD9^−/−^  *n* = 10. Data are displayed as mean with SD. Immunohistochemistry, scale bar: 100 µm (**A**,**B**,**D**,**E**,**J**,**K**) and 20 µm (**A**,**B**,**D**,**E**,**J**,**K**, inserts; **G**,**H**).

TMEV primarily targets the hippocampus of mice and causes polioencephalitis in C57BL/6 mice during acute infection [15]. Histology of infected mice revealed the presence of inflammatory responses within the hippocampus characterized by mononuclear infiltrates and gliosis associated with neuronal loss of the stratum pyramidale. A significantly increased severity of hippocampal damage, as determined by semiquantitative scoring, was found in CARD9^−/−^ mice compared to WT mice at 7 dpi (*p* = 0.03; Figure 1A–C). Accordingly, an increased loss of NeuN^+^ mature neurons in the stratum pyramidale was found in CARD9^−/−^ mice compared to WT mice at 7 dpi by densitometry (*p* = 0.05; Figure 1D–F). To detect more subtle brain lesions, labeling of DCX^+^ neuronal progenitor cells and β-APP, as a marker for damaged axons, was performed. Here, a significant reduction in DCX^+^ cells were observed in the hippocampal dentate gyrus of CARD9^−/−^ mice compared to WT mice at 7 dpi (*p* < 0.01) and 14 dpi (*p* = 0.01; Figure 1G–I), indicative of disturbed neurogenesis. Moreover, a significant accumulation of swollen β-APP^+^ axons preferentially in the cornu ammonis region CA1 of CARD9^−/−^ mice compared to WT mice was detected at 7 dpi (*p* = 0.03) and 14 dpi (*p* = 0.03; Figure 1J–L).

Conclusively, data show that CARD9 deficiency enhances hippocampal damage with increased neuronal loss, axonopathy and disturbed neurogenesis following acute TMEV infection.

### 2.2. CARD9 Deficiency Transiently Increases the Viral Burden but Does Not Prevent Viral Clearance

**Figure 2:**
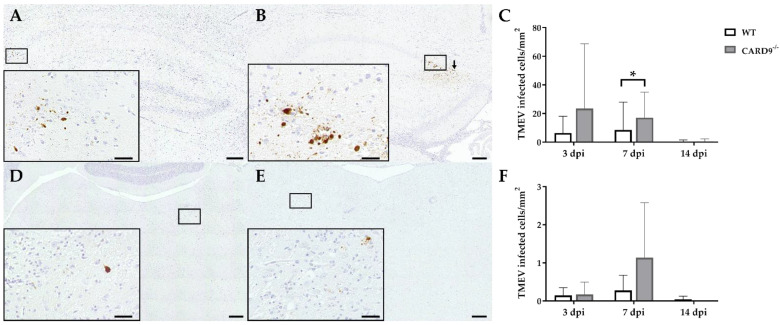
Quantification of Theiler‘s murine encephalomyelitis virus (TMEV)-infected cells in C57BL/6 wild-type (WT) mice and CARD9^−/−^ mice. TMEV-infected cells in the hippocampus of (**A**) a WT and (**B**) a CARD9^−/−^ mouse at 7 days post infection (dpi). (**B**) TMEV antigen (arrows) primarily in pyknotic hippocampal neurons of a CARD9^−/−^ mouse at 7 dpi. (**C**) Quantification of TMEV-infected cells in the hippocampus. TMEV-infected cells in the brainstem of (**D**) a WT and (**E**) a CARD9^−/−^ mouse at 7 dpi. (**F**) Quantification of TMEV-infected cells in the brainstem. (**A**,**B**,**D**,**E**) TMEV-specific immunohistochemistry. * Significant difference between WT and CARD9^−/−^ mice (*p* ≤ 0.05; Mann–Whitney *U*-test). Three dpi: WT *n* = 10, CARD9^−/−^  *n* = 10; 7 dpi: WT *n* = 10, CARD9^−/−^  *n* = 9; 14 dpi: WT *n* = 10, CARD9^−/−^  *n* = 10. Data are displayed as mean with SD. Immunohistochemistry, scale bar: 100 µm (**A**,**B**,**D**,**E**) and 20 µm (**A**,**B**,**D**,**E**).

TMEV antigen was found predominantly in hippocampal pyramidal neurons of the CA1 regions in both CARD9^−/−^ and WT mice following infection, as demonstrated by immunohistochemistry. Quantification revealed a significant increase in TMEV-infected cells in the hippocampus of CARD9^−/−^ mice compared to WT mice at 7 dpi (*p* = 0.05). At 14 dpi, only a few TMEV-infected cells were detected in the forebrain in CARD9^−/−^ mice and WT mice, indicating virus elimination in both groups (Figure 2A–C). Few TMEV-infected cells were found in the brain stem of infected mice without significant differences observed between CARD9^−/−^ and WT mice (Figure 2D–F). In addition, only a few TMEV-infected cells were found in the spinal cord of animals (Figure S2) without differences observed between both groups. No infected cells were found in the cerebellum. Using RT-qPCR, an increase in TMEV-RNA levels were only detected by trend (*p* = 0.07) in the brain of CARD9^−/−^ mice at 3 dpi, and no differences of viral RNA levels in the brain of CARD9^−/−^ and WT mice were detected at 7 and 14 dpi (Figure S3).

In conclusion, data reveal that CARD9 deficiency has an only limited effect on the viral brain load during the acute phase of TMEV infection, since it transiently increases the virus brain load but does not affect viral clearance and spread within the CNS.

### 2.3. Hippocampal Damage in CARD9^−/−^ Mice Is Associated with an Increased T Cell, B Cell, Regulatory T Cell and M2-Type Myeloid Cell Infiltration

**Figure 3:**
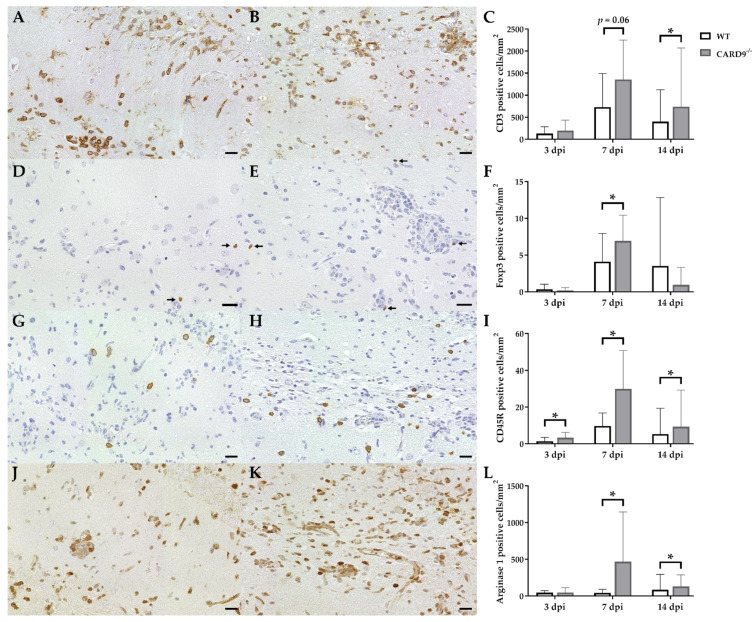
Phenotyping of immune cells in the hippocampus of C57BL/6 wild-type (WT) mice and CARD9^−/−^ mice following Theiler’s murine encephalomyelitis virus (TMEV) infection. CD3-specific immunostaining in the hippocampus of (**A**) a WT mouse and (**B**) a CARD9^−/−^ mouse at 7 days post infection (dpi), which shows a prominent infiltration of CD3^+^ T cells (arrows) in CARD9^−/−^ mice. (**C**) Quantification of CD3^+^ T cells in the hippocampus. (**D**) Image of few Foxp3^+^ regulatory T cells (arrow) in a WT mouse at 7 dpi. (**E**) Increased numbers of Foxp3^+^ cells (arrows) in a CARD9^−/−^ mouse at 7 dpi. (**F**) Quantification of Foxp3^+^ regulatory T cells in the hippocampus. (**G**) Low numbers of CD45R^+^ B cells in a WT mouse at 7 dpi. (**H**) Increase in CD45R^+^ B cells in the hippocampus of a CARD9^−/−^ mice at 7 dpi. (**I**) Quantification of CD45R^+^ B cells in the hippocampus. (**J**) Image of arginase 1^+^ M2-type macrophages/microglia in the hippocampus of a WT mouse at 7 dpi. (**K**) Increase in arginase 1^+^ M2-type macrophages/microglia in a CARD9^−/−^ mice at 7 dpi. (**L**) Quantification of arginase 1^+^ cells in the hippocampus. * Significant difference between WT and CARD9^−/−^ mice (*p* ≤ 0.05; Mann–Whitney *U*-test). Three dpi: WT *n* = 10, CARD9^−/−^  *n* = 10; 7 dpi: WT *n* = 10, CARD9^−/−^  *n* = 9; 14 dpi: WT *n* = 10, CARD9^−/−^  *n* = 10. Data are displayed as mean with SD. Immunohistochemistry, scale bar: 20 µm (**A**,**B**,**D**,**E**,**G**,**H**,**J**,**K**).

Immunohistochemistry was performed to phenotype immune cell sequestration in the hippocampus. Infected CARD9^−/−^ mice showed a significantly enhanced recruitment of CD3^+^ T cells in the hippocampus at 14 dpi (*p* = 0.02) compared to infected WT mice (Figure 3A–C).

Compared to CD3^+^ T cells, only a few Foxp3^+^ regulatory T cells were found in hippocampi of infected mice. At 7 dpi, elevated numbers of Foxp3^+^ cells were found in CARD9^−/−^ mice (*p* = 0.05; Figure 3D–F), while no significant differences of infiltrating granzyme B^+^ cells (effector T cells) between CARD9^−/−^ and WT mice were found in the hippocampus at any time point. CARD9^−/−^ mice exhibit a significantly increased expansion of CD45R^+^ B cells in the hippocampus compared to WT mice at all investigated time points (3 dpi, *p* = 0.03; 7 dpi, *p* = 0.01; 14 dpi, *p* = 0.02; Figure 3G–I). No significant changes in CD107b^+^ microglia/macrophage numbers were observed between CARD9^−/−^ and WT mice at any time point post infection. However, increased numbers of arginase 1^+^ myeloid cells (M2-type macrophages/microglia) were present in the hippocampus of CARD9^−/−^ mice compared to WT mice at 7 dpi (*p* = 0.03) and 14 dpi (*p* = 0.03; Figure 3J–L).

Conclusively, data indicate that the enhanced hippocampal damage and neuroinflammation in CARD9^−/−^ mice is accompanied by modulatory responses, such as an enhanced M2-polarization of macrophages/microglia and regulatory T cell recruitment.

### 2.4. CARD9^−/−^ Mice Develop Early Pro-Inflammatory Cytokine Responses in the Brain

**Figure 4:**
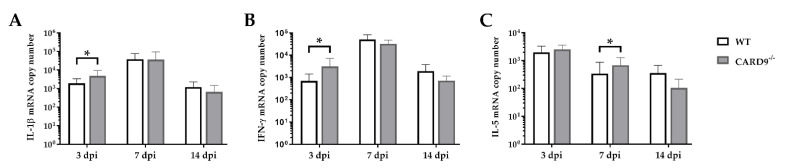
Cytokine mRNA expression in the cerebrum of C57BL/6 wild-type (WT) mice and CARD9^−/−^ mice following Theiler’s murine encephalomyelitis virus (TMEV) infection. Quantification of (**A**) interleukin (IL)-1β, (**B**) interferon (IFN)-γ and (**C**) IL-5 mRNA levels in the cerebrum by reverse transcription quantitative polymerase chain reaction (RT-qPCR). * Significant difference between WT and CARD9^−/−^ mice (*p* ≤ 0.05; Mann–Whitney *U*-test). Three dpi: WT *n* = 10, CARD9^−/−^  *n* = 10; 7 dpi: WT *n* = 10, CARD9^−/−^   *n* = 9; 14 dpi: WT *n* = 10, CARD9^−/−^  *n* = 10. Data are displayed as mean with SD.

Cytokine expression analyses were performed to characterize inflammatory responses in the brain in more detail. RT-qPCR revealed a significant increase in IL-1β (*p* = 0.02) and IFN-γ (*p* = 0.03) in the brain of CARD9^−/−^ mice compared to WT mice following TMEV infection at 3 dpi (Figure 4A,B). At 7 dpi, a significantly increased transcription of IL-5 (*p* = 0.05) was found in the brain of infected CARD9^−/−^ mice compared to WT controls (Figure 4C). Gene expression of IL-1α, IL-4, IL-6, IL-10, TGF-β1 and TNF-α did not differ between groups at any investigated time point post infection (Figure S4). Between non-infected CARD9^−/−^ mice and WT mice, no differences were found in the brain for all investigated cytokines (Figure S6).

Data show that CARD9 deficiency is able to alter cytokine expression in the brain with temporarily increased pro-inflammatory cytokine responses early after infection. During the infection course, expression of IL-5 might be associated with compensatory mechanisms in response to hippocampal alteration in CARD9^−/−^ mice.

### 2.5. CARD9 Deficiency Has a Limited Effect on Initial CD8^+^ T Cell Responses

**Figure 5:**
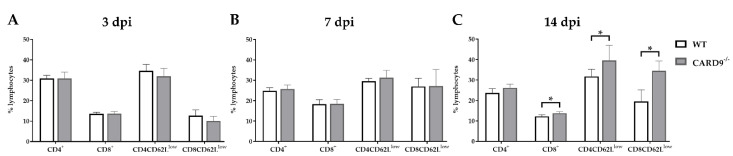
Flow cytometric analysis of CD4^+^ and CD8^+^ T cell responses. Quantification of CD4^+^, CD8^+^, CD4^+^CD62L^low^ and CD8^+^CD62L^low^ T cells in the spleen of C57BL/6 wild-type (WT) and CARD9^−/−^ mice following Theiler’s murine encephalomyelitis virus (TMEV) infection at (**A**) 3 days post infection (dpi), (**B**) 7 dpi and (**C**) 14 dpi. * Significant difference between WT and CARD9^−/−^ mice (*p* ≤ 0.05; Mann–Whitney *U*-test). Three dpi: WT *n* = 10, CARD9^−/−^  *n* = 10; 7 dpi: WT *n* = 10, CARD9^−/−^  *n* = 9; 14 dpi: WT *n* = 10, CARD9^−/−^  *n* = 10. Data are displayed as mean with SD.

Timely peripheral activation and priming of naïve T cells in lymphoid organs during the early phase was shown to be crucial for mounting robust antiviral responses in the TMEV model [40,41]. To determine whether CARD9 deficiency affects peripheral T cell activation, flow cytometric analysis of spleen tissue has been performed. In agreement with the observed limited effect of CARD9 on the viral brain load, analyses revealed no variations of T cell activation marker expression between CARD9^−/−^ mice and WT mice at 3 and 7 dpi (Figure 5A,B). Increased frequencies of splenic CD8^+^ T cells, CD4^+^CD62L^low^ and CD8^+^CD62^low^ T cells were detected in CARD9^−/−^ mice compared to WT mice at 14 dpi (Figure 5C), probably associated with hippocampal damage and the prolonged CD3^+^ T cell responses in the brain determined by immunohistochemistry. Non-infected CARD9^−/−^ mice showed increased frequencies of splenic CD4^+^ and CD8^+^ cells compared to WT mice, while no differences in CD4^+^CD62L^low^ and CD8^+^CD62^low^ T cell frequencies were found between both groups under non-infectious conditions (Figure S7).

Conclusively, data indicate that CARD9 deficiency does not significantly affect T cell priming in peripheral lymphoid organs during the initial phase of TMEV infection.

### 2.6. CARD9 Deficiency Does Not Affect Antigen Presenting Cells Ability to Prime CD8^+^ T Cells Following Theiler’s Murine Encephalomyelitis Virus Infection In Vitro

**Figure 6:**
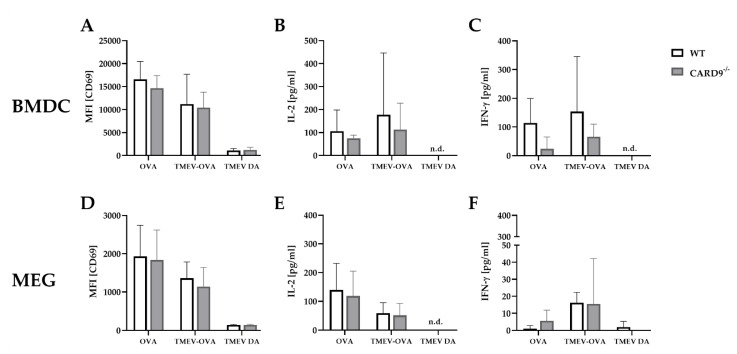
In vitro CD8^+^ T cell response following incubation with ovalbumin (OVA), TMEV-OVA and TMEV DA of (**A**–**C**) bone-marrow-derived dendritic cells (BMDCs) or (**D**,**F**) microglia-enriched glial cell mixtures (MEGs). The mean fluorescence intensity (MFI) of activation marker CD69 gated on CD8^+^ T cells derived from OT-I mice after co-cultivation with (**A**) BMDCs or (**D**) MEGs was analyzed by flow cytometry. ELISA-based analysis of interleukin 2 (IL-2) and interferon γ (IFN-γ) cytokine production following co-cultivation with (**B**,**C**) BMDCs or (**E**,**F**) MEGs. Statistical analysis was done by Mann–Whitney *U*-test. Data are displayed as mean with SD. n.d. = not detectable.

To support the finding of a limited effect of CARD9 deficiency on T cell priming and to test the direct effect of CARD9 deficiency upon antigen presentation, BMDC as professional antigen presenting cells (APC) were utilized in an in vitro stimulation assay. WT and CARD9^−/−^ BMDC were incubated with live TMEV DA at a MOI 200, which had no visible effect on BMDC viability, but is necessary to induce adequate APC-specific effector functions. The presence of the virus led to BMDC activation, indicated by an upregulation of the surface marker CD86 and MHC I and an increased release of inflammatory cytokines IL-6 and IFN-β (Figure S5). No differences in activation marker expression and cytokine production were observed between WT and CARD9^−/−^ BMDCs following activation with TMEV. To analyze the impact of CARD9 deficiency on early TMEV-mediated T cell responses in vitro, a BMDC/T cell and MEG/T cell co-cultivation system was used. OT-I T cells that specifically recognize the OVA_257–264_ peptide presented via the MHC I molecule H2-K^b^ were co-cultured with BMDCs or MEGs that had been exposed to TMEV-OVA. In this setting, non-OVA-expressing TMEV DA was utilized as a negative control. To evaluate CD8^+^ T cell activation, expression of the T cell activation marker CD69 was analyzed by flow cytometry (Figure 6A,D), and cytokine release was measured by ELISA (Figure 6B,C,E,F). Both co-culture systems revealed a TMEV-OVA-mediated increase in CD8^+^ T cell activation compared to non-OVA expressing TMEV DA exposed cells, indicated by upregulated levels of CD69 as well as an elevated release of IL-2 and IFN-γ (Figure 6B,C,E,F). However, no difference in CD8^+^ T cell responses between the WT and CARD9^−/−^ group was observed, neither when BMDCs nor when MEGs were used in the co-culture system. Furthermore, the overall CD8^+^ T cell response following TMEV-OVA incubation of MEGs was comparably low, suggesting that the potential of antigen processing and presentation of microglia is limited compared to BMDCs in vitro.

In summary, these experiments reveal that TMEV stimulation initiates CD8^+^ T cell priming by APC-mediated antigen presentation. However, CARD9 deficiency does not alter CD8^+^ T cell priming in vitro. Combined with the unaltered early T cell responses found in vivo (Figure 6A,B), CARD9 signaling most likely does not substantially contribute to the development of antiviral CD8^+^ T cell responses against TMEV.

## 3. Discussion

CARD9 is an adaptor protein, which is involved in the Syk-dependent signaling cascade of CLRs bearing a hemITAM or being linked to an ITAM such as Dectin-1, Dectin-2 and Mincle [42]. These CLRs are most prominently involved in the recognition of fungal and bacterial pathogens, whereas their contribution to viral recognition is largely unknown [43]. Hence, to investigate the contribution of CARD9-dependent signaling of activating hemITAM-bearing or ITAM-coupled CLRs to virus control and neuropathology, CARD9^−/−^ mice were utilized in a murine TMEV infection model.

The present study reveals a rather limited role of CARD9 for the initiation of antiviral responses following TMEV infection in C57BL/6 mice. Nonetheless, an enhanced severity of polioencephalitis in CARD9^−/−^ mice supports the view that intact CARD9 signaling contributes to reducing hippocampal damage during acute neurotropic virus infection. Initiation of robust CD8-mediated cytotoxicity early after infection accounts for TMEV elimination from the CNS in C57BL/6 mice [15]. CARD9 deficiency transiently increases hippocampal infection but seems to be inefficient for delaying viral elimination. Moreover, CARD9 deficiency does not enhance viral spread to the brain stem and spinal cord, as observed in TMEV-infected SJL mice and CD8-deficient C57BL/6 mice [44,45,46]. As shown, CARD9 deficiency is not able to reduce the recruitment of granzyme B^+^ effector cells in the hippocampus of TMEV-infected mice. Furthermore, adaptor protein signaling does not sustainably affect peripheral T cell priming in the TMEV model, since splenic CD4^+^ and CD8^+^ T cells of CARD9^−/−^ mice showed unaltered expressions of activation markers during the initial phase (3 and 7 dpi). Previously, CARD9^−/−^ mice were shown to display normal T cell proliferative capacities and an upregulation of surface activation markers similar to wild-type mice after T cell receptor stimulation, supporting the view that CARD9 is dispensable for T cell development and activation [47].

Performed co-culture experiments using DCs further support the idea that CARD9 deficiency does not substantially affect the TMEV-specific T cell priming capacity of antigen-presenting cells. Similar to the present in vitro experiments, CARD9 deficiency does not affect IFN-α and/or IFN-β production by DCs following influenza virus or vesicular stomatitis virus (VSV) infection, respectively. Moreover, CARD9^−/−^ mice show no altered type I interferon responses following influenza virus or VSV infection in vivo, indicating that CARD9-dependent signals might be important in shaping T cell responses to viruses primarily at later stages of infection but do not mediate immediate early antiviral protection [27,36,38].

Results of the present study are in agreement with the findings in influenza virus infection, showing that CD8^+^ T cell responses and viral clearance is not compromised in CARD9^−/−^ mice [38]. Moreover, La Crosse virus-infected CARD9^−/−^ DCs show no reduction in the virus load compared to wild-type cells in vitro, suggesting a limited role of the adaptor protein in early antiviral responses [32]. The present data seemingly contrast with observations in neuroinvasive fungal diseases (candidiasis) in CARD9^−/−^ mice and humans with inherited CARD9 deficiency, showing that the adaptor protein critically controls brain infection [48,49,50,51]. However, it should be noted that the protective effect of CARD9 in fungal diseases is mediated primarily by the recruitment of neutrophils and increased neutrophilic responses in the CNS, which play a negligible role for TMEV elimination. The function of CARD9 in infectious diseases seems to be clearly context-dependent and varies among different pathogens, disease phases and affected organ systems [27].

CARD9^−/−^ mice showed a limited but significant increase in neuronal loss, axonopathy and disturbed neurogenesis in the hippocampus following TMEV infection. Axonal damage in infected CARD9^−/−^ mice is supposed to be a consequence of virus-induced cell damage and increased neuroinflammation, respectively. Axonopathies contribute to neurological disability and represent a potential early trigger for immune-mediated tissue damage [52,53]. However, because TMEV is transported along axons in the brain, axon damage in C57BL/6 mice is also regarded as a mechanism that limits virus dissemination in the CNS [54]. However, it remains speculative whether hippocampal axonopathy might have contributed to the prevention of TMEV spread to the brain stem and spinal cord in CARD9^−/−^ mice. Of note, previous studies did not observe an impact of acute TMEV infection (14 dpi) on hippocampal neurogenesis and the number of DCX^+^ neuronal progenitor cells in the dentate gyrus of C57BL/6 mice [55]. Thus, reduced numbers of hippocampal DCX^+^ cells in CARD9^−/−^ mice indicate a protective effect of intact CARD9 signaling upon hippocampal neurogenesis. Hippocampal changes following subclinical infection have been shown to impair memory functions, as observed in murine tick-borne encephalitis virus models [8]. Self-limiting acute viral encephalitis has also been shown to elicit long-term neurologic deficits in human patients, including memory problems and an increased risk of epilepsy [3,4]. The relevance of hippocampal pathology for cognitive functions and its long-term consequences in CARD9^−/−^ mice remain elusive and should be investigated in future studies.

Although the adaptor protein mediates pro-inflammatory cytokine responses via NF-κB activation in myeloid cells, CARD9 deficiency is not able to control inflammatory and neurotoxic responses in the brain during TMEV infection. Even an increased expression of IFN-γ and IL-1β was observed in CARD9^−/−^ mice during the early infection phase (3 dpi), indicating the presence of a CARD9-independent activation of cytokine responses. The pro-inflammatory cytokines IFN-γ and IL-1β generally appear in the acute phase of virus infection. IFN-γ is produced primarily by activated T cells and natural killer (NK) cells, as well as by NKT cells, B cells and antigen presenting cells, while IL-1β derives from activated macrophages and microglia [56,57,58]. Similar to the present findings, IFN-γ production is increased in lung tissues of CARD9^−/−^ mice challenged with influenza virus [38]. In addition, CARD9^−/−^ mice exhibit an early increase in IFN-γ blood levels in response to *Listeria monocytogenes* infection [35]. IFN-γ and IL-1β are crucial for the initiation of protective immunity in many viral diseases but also responsible for immune-mediated tissue damage [59,60]. In TMEV infection, cytokine balance needs to be tightly controlled, since excessive IL-1β levels have been shown to impair protective immunity and lead to persistent infection in C57BL/6 mice [61,62]. IL-1β has been reported to exacerbate neurodegeneration and plays a pivotal role for suppressing neurogenesis in the hippocampus [63,64,65]. Moreover, IL-1β is responsible for excitotoxicity and neuronal damage in models of viral encephalomyelitis caused by neuroadapted *Sindbis virus* [66]. Similar to the present findings, CARD9^−/−^ mice exhibit increased splenic IL-1β expressions following systemic *Salmonella* infection. Here, the adaptor protein negatively regulates cytokine expression by fine-tuning pro-IL-1β expression in macrophages following bacterial infection, contrasting its role in fungal infection in which CARD9 primarily drives pro-inflammatory cytokine responses [67].

Hippocampal damage in CARD9^−/−^ mice is associated with increased numbers of alternatively activated M2-type cells and Foxp3^+^ regulatory T cells. M2-type myeloid cells favor Th2 immune responses, which might account for increased IL-5 mRNA levels and B cell responses found in the brain of CARD9^−/−^ mice following TMEV infection. CARD9 has been shown to be essential for M1-polarization of myeloid cells [68,69], supporting the view that the loss of CARD9 enhances M2-polarization following TMEV infection. M2-polarization of macrophages/microglia and sequestration of regulatory T cells represent compensatory mechanisms that facilitate tissue repair in response to brain injury. However, M2-type cells also negatively affect the development of antiviral immunity [70]. Thus, one can speculate that altered immune responses related to M2-type microglia/macrophages have contributed to the transiently increased virus load in the brain of CARD9^−/−^ mice in the present study. In general, regulatory T cells are also able to dampen antiviral responses during TMEV infection. However, due to the low number of infiltrating Foxp3^+^ cells, their impact upon local antiviral responses in the CNS remains unclear in the present study [44,71,72].

Conclusively, the present study indicates that CARD9 is dispensable for the initiation of CD8-mediated antiviral responses and TMEV elimination in C57BL/6 mice. However, CARD9 signaling has the ability to modulate inflammatory responses following acute neurotropic virus infection, thereby reducing hippocampal damage. The underlying mechanism remains unclear, and further studies are clearly needed to obtain detailed insights into the regulation of CARD9 signaling and the respective CLRs involved in TMEV infection and other infectious disorders of the CNS.

## 4. Materials and Methods

### 4.1. Study Design

Female CARD9^−/−^ mice (*n* = 29) and C57BL/6 mice (WT; *n*= 30) were infected intracerebrally with the Daniels strain of TMEV (TMEV DA) [24,73]. Non-infected CARD9^−/−^ mice (*n* = 5) and C57BL/6 mice (*n*= 5) were used as controls. Mice were kept in individually ventilated cages under standardized conditions with ad libitum access to standard rodent feed and water. TMEV DA propagation for intracerebral infection was performed on BHK-21 cells with a MOI of 1.0. The virus titer was determined via plaque assays on L-cells as described previously [74,75,76,77]. Intracerebral infection with the TMEV DA was performed as described previously [23,78,79]. General anesthesia was induced with medetomidine (1 mg/kg, Domitor^®^) and ketamine (100 mg/kg) [44]. Twenty microliters of virus solution (1 × 10^5^ plaque forming units) was injected into the right cerebral hemisphere by a free-hand method using a 26G cannula and a 50 μL syringe (Hamilton, Bonaduz, Switzerland). The depth of entry was limited to 2 mm by using a stop collar. Mice were allowed to recover from anesthesia, before being returned to their cages. Necropsies of infected animals were performed at 3, 7 and 14 days post infection (dpi). Therefore, animals were deeply anesthetized and transcardially perfused with phosphate buffered saline (PBS). The caudal part of the left cerebrum (contralateral to injection site), cerebellum, brain stem and spinal cord were formalin fixed and paraffin embedded (FFPE), and the rostral part of the left cerebrum was snap-frozen at −80 °C. Histologic evaluation and immunohistochemistry of hippocampi were performed on coronal sections at Bregma −1.46 to −1.82 [80]. Spleen tissue was collected for flow cytometry.

### 4.2. Histological and Immunohistochemical Staining

Histologic grading of hippocampal damage was determined on three serial hematoxylin and eosin (HE)-stained FFPE sections. The integrity of pyramidal neurons in the hippocampus was evaluated by applying a 5-point semiquantitative scoring system, modified from Rattka et al.: 0 = no obvious damage; 1 = loss of single neurons (affecting <10% of pyramidal neurons); 2 = areas with segmental neuronal loss (affecting <20% of pyramidal neurons); 3 = one or more areas with segmental neuronal loss (affecting 20–50% of pyramidal neurons); 4 = areas with segmental neuronal loss (affecting >50% of pyramidal neurons) [81].

Infiltrating immune cells, astroglial responses, axonal damage, neuronal integrity, hippocampal neurogenesis and TMEV infection were quantified by immunohistochemistry on three serial tissue sections each. The antibodies utilized with their specificities and staining procedures are listed in Table S1. Visualization of antibody binding was achieved by application of avidin biotin peroxidase complex together with 3,3’-diaminobenzidine as chromogen and counterstaining with Mayer’s hematoxylin [82,83]. Morphometric analyses of hippocampi were conducted using the fluorescence microscope BZ-9000 BIOREVO with the BZ-II Analyzer software (Keyence Corporation, Osaka, Japan). Each stained slide was digitalized, and sizes of hippocampi (mm^2^) were determined by manually circumscribing the hippocampal area using the hybrid cell count function of the software (Figure S1). The size of lesions (neuronal loss) within pyramidal layers was morphometrically assessed in NeuN-stained sections using brightness intensity extraction of the software. Data were expressed as percentage of NeuN immunopositive area per total area of the hippocampus (NeuN immunopositive area %). Similarly, the amount of GFAP immunostaining per hippocampus was quantified morphometrically. The absolute numbers of labelled cells within the hippocampus were manually counted by light microscopy and expressed as labelled cells per total area of the hippocampus (cells/mm^2^) for B cells (CD45R), T cells (CD3), regulatory T cells (Foxp3), granzyme B expressing effector cells, macrophages/microglia (CD107b) and M2-type macrophages/microglia (arginase 1). In addition, β-amyloid precursor protein (β-APP)^+^ axons were counted manually within the hippocampus. Doublecortin (DCX) expressing neuronal precursor cells were determined as labelled cells/mm^2^ within the dentate gyrus of the hippocampus. Moreover, the quantity of TMEV-infected cells within different regions of the CNS (hippocampus, brain stem, cerebellum, spinal cord) was assessed by counting stained cells.

### 4.3. Real-Time PCR

For subsequent RT-qPCR analyses, RNA was isolated from snap-frozen cerebral tissue and translated into cDNA. Therefore, specimens were homogenized in QIAzol^®^ lysis reagent (Qiagen, Hilden, Germany) and isolation was performed with the RNeasy^®^ Lipid tissue Mini Kit (Qiagen, Hilden, Germany) according to the manufacturer’s protocol. RNA purity and amount were determined by using a spectrophotometer (Thermo Fisher Scientific, Braunschweig, Germany, Multiskan^TM^ GO microplate spectrophotometer, µDrop^TM^ plate, SkanIt^TM^ software version 5.0.0.42). Translation into cDNA was achieved by using the Omniscript^®^ Reverse Transcription Kit (Qiagen, Hilden, Germany), RNaseOUT^TM^ Recombinant Ribonuclease Inhibitor (Invitrogen^TM^) and random primers (Promega Corporation, Walldorf, Germany).

RT-qPCR was performed using the AriaMx Real-Time PCR System (Agilent Technologies, Agilent Aria software v1.71) and Brilliant III Ultra-Fast SYBR^®^ Green QPCR Mastermix (Agilent Technologies, Waldbronn, Germany). Viral RNA load and mRNA expression of cytokines within the cerebrum were determined (Table S2). By applying the standard curve method (standards ranging from 10^8^–10^2^ copies/µL) and measuring three housekeeping genes (β-actin, glyceraldehyde 3-phosphate dehydrogenase (GAPDH), hypoxanthine-guanine phosphoribosyltransferase (HPRT)) with subsequent normalization (geNorm software 3.4), copy numbers were calculated. Reaction specificities were proved by melting curve analysis [84,85].

### 4.4. Flow Cytometric Immunophenotyping of Murine Splenocytes

Splenocytes were isolated by mechanically flushing freshly removed spleens with a syringe and 1x PBS to obtain a single cell suspension. Red blood cells (RBC) within cell suspensions were lysed using RBC lysis buffer (10% 100 mM Tris-HCl [Tris-(hydroxymethyl)-aminomethanhydrochloride], 90% 160 mM NH_4_Cl [ammonium chloride]). Cells were incubated with rat-anti CD16/32 monoclonal antibody (1:100) and stained with monoclonal anti-mouse antibodies listed in Table S3. Afterwards, cells were incubated with 1% paraformaldehyde (PFA). For flow cytometry, the Attune NxT flow cytometer (Thermo Fisher Scientific) was used, and data analysis was conducted with FlowJo (FloJo LLC).

### 4.5. In Vitro T Cell Co-Cultivation

For in vitro T cell co-cultivation assays with both adult-microglia-enriched glial cell mixtures (MEG) and bone-marrow-derived dendritic cells (BMDC), TMEV DA and ovalbumin peptide (OVA_244–260_)-expressing TMEV DA XhoI-OVA8 (TMEV-OVA) that includes the H-2K^b^-restricted CD8^+^ T cell epitope SIINFEKL were used [86,87]. Virus propagation and titer determination were performed as previously described, using a MOI of 0.1 for TMEV-OVA.

MEGs were isolated as described by Prajeeth et al. (2014) [88] with slight modifications. Briefly, brains of WT and CARD9^−/−^ mice were dissected and dissociated by squashing the tissue with the top end of a syringe in a 6-well plate containing a digestion solution (HBSS, 1 mg/mL collagenase D, 5 U/mL DNase I). After incubation for 10 min at 37 °C, brains were gently dissociated by pipetting up and down. Subsequently, 40% Percoll centrifugation (10 min, 350× *g*, 18 °C) and RBC lysis were performed. To evaluate the proportion of microglia within MEG, cells were blocked with anti-mouse CD16/32, stained with anti-mouse CD11b-PE and anti-mouse CD45-APC and fixed in 1% PFA. Flow cytometry was performed using an Attune NxT flow cytometer. Data analysis was conducted with FlowJo. The purity of microglia (CD11b^+^/CD45^low+^) within MEG used for co-culture experiments ranged from 50% to 70% for cell suspensions obtained from both WT and CARD9^−/−^ mice.

Following the isolation of MEGs, cells were seeded with 4 × 10^4^ cells/well in a 96-well U-bottom plate in culture medium (IMDM medium, 10% FCS, 2 mM L-glutamine, 100 U/mL penicillin 100 µg/mL streptomycin) and stimulated with EndoGrade^®^ ovalbumin (0.3 mg/mL) or TMEV-OVA (MOI 200) for 22 h at 37 °C. T cells were isolated from spleens of 8- to 12-week-old OT-I transgenic mice by magnetic activated cell sorting (Pan T Cell Isolation Kit II mouse, Miltenyi Biotec, Bergisch Gladbach, Germany). An amount of 1 × 10^5^ cells/well of the purified T cells was added to the glial cell mixture and co-cultured at 37 °C. After 48 h, supernatants were harvested and cytokine concentrations of IL-2 and IFN-γ were analyzed by ELISA (murine IL-2 and IFN-γ Standard ABTS ELISA Development Kit, PeproTech, Hamburg, Germany). Co-cultured cells were blocked with anti-mouse CD16/32, stained with anti-mouse CD8a-FITC, CD62L-PE and CD69-APC and fixed in 1% PFA. Flow cytometry was performed using an Attune NxT flow cytometer, and data analysis was conducted with FlowJo software.

Bone marrow cells were isolated from femurs and tibias of WT and CARD9^−/−^ mice and differentiated into BMDCs by cultivation with GM-CSF-containing culture medium (IMDM medium, 10% FCS, 2 mM L-glutamine, 100 U/mL penicillin 100 µg/mL streptomycin, 10% X63-GM-CSF supernatant) for 8 to 10 days at 37 °C. Differentiated BMDCs were seeded in a 96-well U-bottom plate with 2 × 10^4^ cells/well in culture medium, and T cell co-cultivation was performed as described above.

### 4.6. Statistical Analysis

Statistical analyses of group comparisons were carried out by applying multiple Mann–Whitney U-tests for non-normal distributed data (SPSS^®^ for Windows, Version 26, SPSS^®^ Inc., IBM Corp., Ehningen, Germany). A *p*-value ≤ 0.05 was assessed as statistical significance. Graphs were built by using GraphPad Prism software (Version 9.0.0, GraphPad Software Inc., San Diego, USA). Significant differences were represented by asterisks (* ≙ *p* ≤ 0.05, ** ≙ *p* ≤ 0.01).

### 4.7. Ethic Statement

The study was approved and authorized by the Niedersächsisches Landesamt für Verbraucherschutz und Lebensmittelsicherheit (LAVES), Oldenburg, Germany (permission number 33.19-42502-04-16/2225). All animal experiments were carried out in accordance with the German law for animal protection and the Directive 2010/63/EU of the European Parliament and of the Council on the protection of animals used for scientific purposes.

## Data Availability

The data presented in this study are available on request from the corresponding author.

## Supplementary Materials

The following are available online at https://www.mdpi.com/article/10.3390/ijms22136982/s1.
