# Supplementary material for: CARD9 Deficiency Increases Hippocampal Injury Following Acute Neurotropic Picornavirus Infection but Does Not Affect Pathogen Elimination
Source: Int J Mol Sci. 2021 Jun 29;22(13):6982. doi: 10.3390/ijms22136982 (PMC8268812; doi:10.3390/ijms22136982)
Supplement: Supplementary file 1 [file ijms-22-06982-s001.zip › ijms-1227378-supplementary.pdf]

International Journal of *Molecular Sciences*

MDPI

Supplementary Materials

CARD9 deficiency increases hippocampal injury following acute neurotropic picornavirus infection but does not affect pathogen elimination

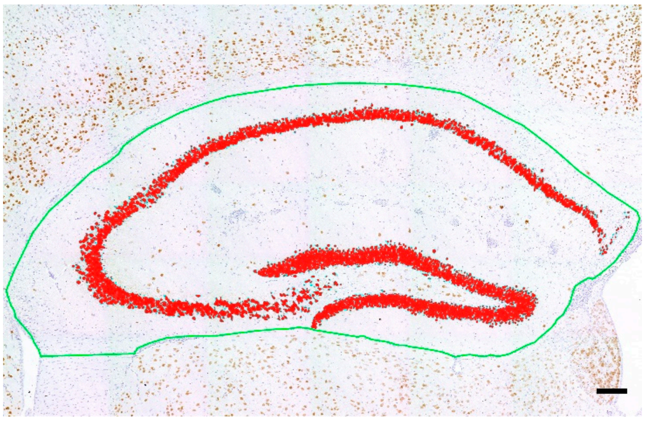

Figure S1. Densitometric analysis using the BZ-II Analyzer software (Keyence Corporation, Osaka, Japan). Representative image of a digitalized slide of a TMEV-infected wild type mouse at 7 days post infection (dpi), showing neuronal nuclear antigen (NeuN)-positive area (red area) in the cornu ammonis (CA) and dentate gyrus regions (brightness intensity extraction). The entire hippocampal area is determined by circumscribing the hippocampal area using the hybrid cell count function of the software (green line). NeuN-specific immunohistochemistry, scale bar: 100 µm.

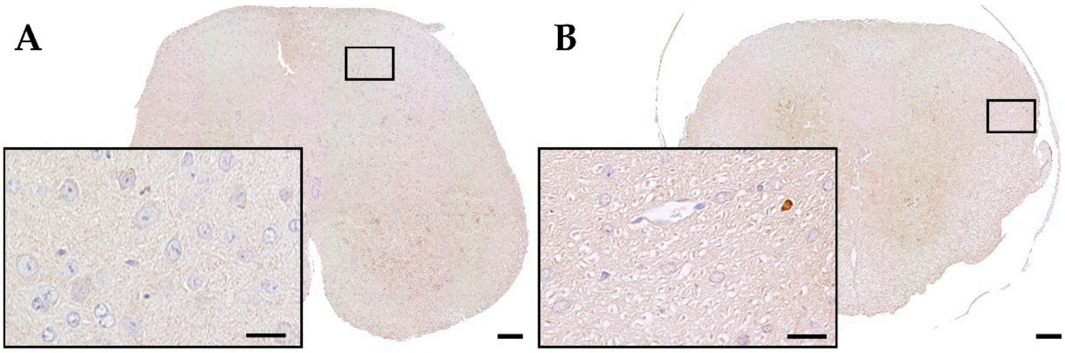

Figure S2. Detection of Theiler's murine encephalomyelitis virus (TMEV) antigen in the spinal cord of C57BL/6 wild type (WT) and CARD9$^{-/-}$ mice. (**A**) Lack of TMEV-infected cells in spinal tissue of a WT mice at 14 dpi. (**B**) Single TMEV-infected cell in the spinal cord of a CARD9$^{-/-}$ mouse at 14 dpi. The quantification of TMEV-infected cells in murine spinal cords revealed no significant difference between WT and CARD9$^{-/-}$ mice. TMEV-specific immunohistochemistry, scale bar: 100 μm (A-B) and 20 μm (A-B inserts).

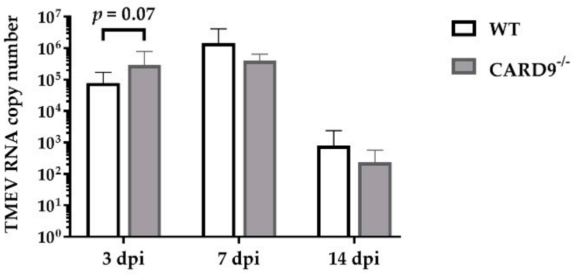

Figure S3. Quantification of Theiler's murine encephalomyelitis virus (TMEV) RNA copy numbers in the cerebrum of C57BL/6 wild type (WT) and CARD9$^{-/-}$ mice. Data were analyzed using Mann-Whitney *U*-test. 3 dpi: WT n=10, CARD9$^{-/-}$ n=10; 7 dpi: WT n=10, CARD9$^{-/-}$ n=9; 14 dpi: WT n=10, CARD9$^{-/-}$ n=10. Graphs show mean values with SD.

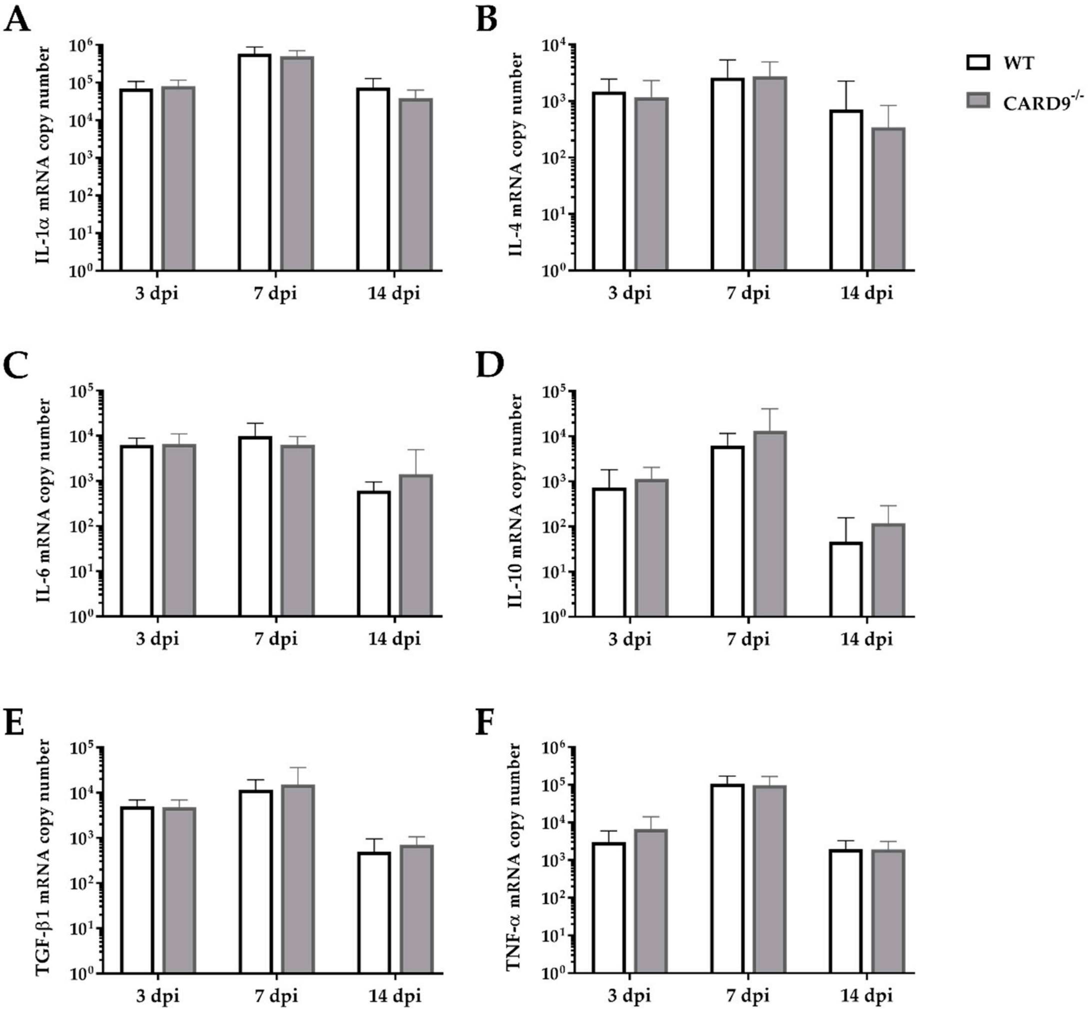

Figure S4. Quantification of cytokine mRNA expression in the cerebrum of TMEV-infected C57BL/6 wild type (WT) and CARD9$^{-/-}$ mice. Quantification of (**A**) interleukin (IL)-1α, (**B**) IL-4, (**C**) IL-6, (**D**) IL-10, (**E**) transforming growth factor (TGF)-β1, and (**F**) tumor necrosis factor (TNF)-α mRNA levels in the cerebrum by reverse transcription quantitative polymerase chain reaction. Difference between WT and CARD9$^{-/-}$ mice were analyzed using the Mann-Whitney *U*-test. 3 dpi: WT n=10, CARD9$^{-/-}$ n=10; 7 dpi: WT n=10, CARD9$^{-/-}$ n=9; 14 dpi: WT n=10, CARD9$^{-/-}$ n=10. Graphs show mean values with SD.

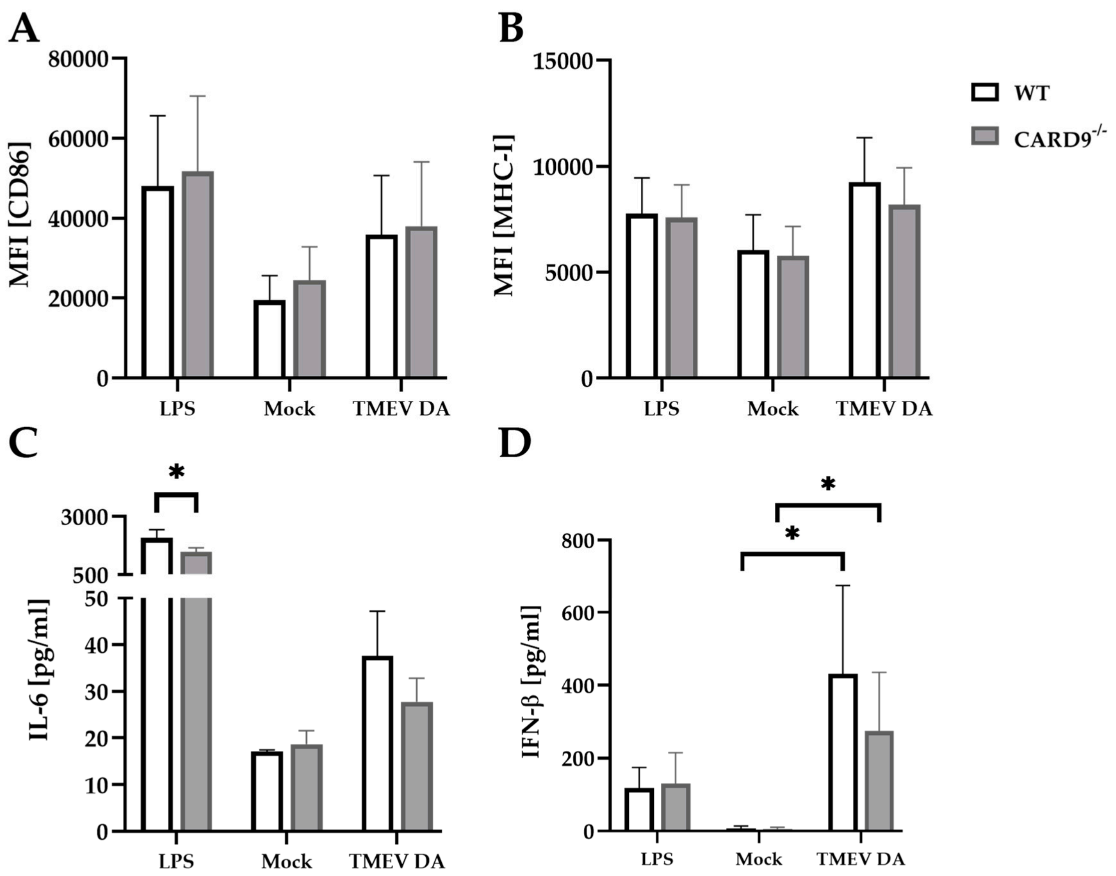

Figure S5. Activation of C57BL/6 wild type (WT) and CARD9$^{-/-}$ bone marrow-derived dendritic cells (BMDCs) following Daniels strain of Theiler's murine encephalomyelitis virus (TMEV DA) stimulation. The mean fluorescence intensity (MFI) of activation markers (**A**) CD86 and (**B**) MHC-I analyzed by flow cytometry. The cytokine productions: (**C**) interleukin (IL)-6 and (**D**) interferon (IFN)-β of stimulated BMDCs analyzed by ELISA. The BMDCs were stimulated by lipopolysaccharide (LPS, positive control), medium (Mock, negative control), or TMEV-DA. * Significant difference between WT and CARD9$^{-/-}$ mice ($p \leq 0.05$; Mann-Whitney *U*-test). 3 dpi: WT n=10, CARD9$^{-/-}$ n=10; 7 dpi: WT n=10, CARD9$^{-/-}$ n=9; 14 dpi: WT n=10, CARD9$^{-/-}$ n=10. Data are displayed as mean with SD.

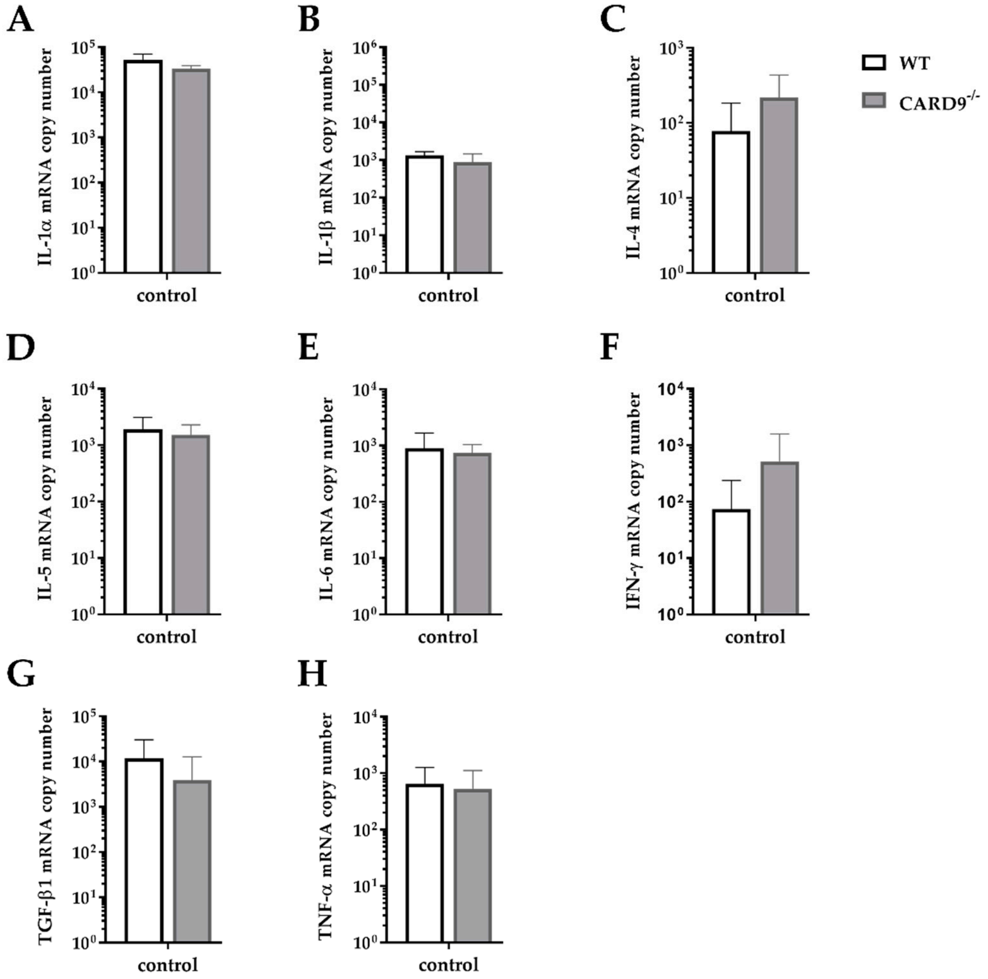

Figure S6. Quantification of cytokine mRNA expression in the cerebrum of non-infected C57BL/6 wild type (WT) and CARD9$^{-/-}$ mice. Quantification of (**A**) interleukin (IL)-1α, (**B**) IL-1β, (**C**) IL-4, (**D**) IL-5, (**E**) IL-6, (**F**) interferon (IFN)-γ, (**G**) transforming growth factor (TGF)-β1, and (**H**) tumor necrosis factor (TNF)-α mRNA levels by reverse transcription quantitative polymerase chain reaction. Difference between WT and CARD9$^{-/-}$ mice were analyzed using the Mann-Whitney *U*-test. IL-10 mRNA was not detectable in WT and CARD9$^{-/-}$ mice. Graphs show mean values with SD. n: 5 WT and 5 CARD9$^{-/-}$ mice.

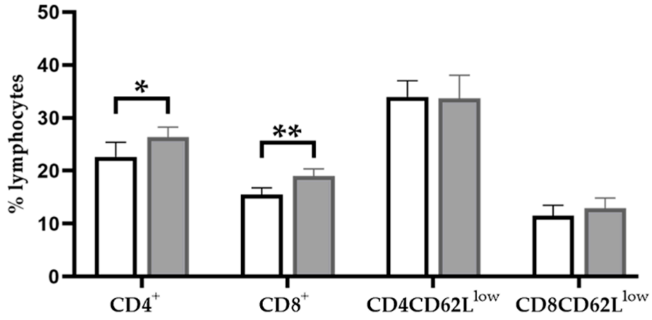

Figure S7. Flow cytometric analysis of $CD4^+$ and $CD8^+$ T cell responses of non-infected C57BL/6 wild type (WT) and $CARD9^{-/-}$ mice. Quantification of $CD4^+$, $CD8^+$, $CD4^+CD62L^{low}$, and $CD8^+CD62L^{low}$ T cells in the spleen of non-infected C57BL/6 wild type (WT) and $CARD9^{-/-}$ mice. (* = $p \leq 0.05$; ** = $p \leq 0.01$, Mann-Whitney *U*-test). Data are shown as mean with SD. n: 5 WT and 5 $CARD9^{-/-}$ mice.

Table S1. Details of antibodies used for immunohistochemistry

Primary antibody	Clonality/clone	Supplier; catalog number	Dilution	Pre-treatment	Blocking serum	Secondary antibody	Specificity
arginase 1	polyclonal	Thermo Fisher Scientific PA5-29645	1:200	HIER	Goat	Goat anti-rabbit	M2-macrophages/ microglia
CD107b	monoclonal M3/84	AbD Serotec MCA2293	1:200	HIER	Rabbit	Rabbit anti-rat	macrophages/ microglia
CD3	polyclonal	DakoCytomation A0452	1:500	HIER	Goat	Goat anti-rabbit	T cells
CD45R	monoclonal RA3-6B2	BD Biosciences 553085	1:1000	HIER	-	-	B cells
DCX	monoclonal E-6	Santa Cruz Biotechnology sc-271390	1:100	HIER	Goat	Goat anti-mouse	neuronal progenitor cells
Foxp3	monoclonal FJK-16s	eBioscience 14-5773-82	1:50	HIER	Rabbit	Rabbit anti-rat	regulatory T cells
GFAP	polyclonal	DakoCytomation Z0334	1:1000	-	Goat	Goat anti-rabbit	astrocytes
GrB	polyclonal	Abcam ab4059	1:400	HIER	Goat	Goat anti-rabbit	effector T cells and NK cells
NeuN	monoclonal A60	Merck/Millipore MAB377	1:1600	HIER	Goat	Goat anti-mouse	neurons
TMEV	polyclonal	Kummerfeld et al. 2009	1:2000	-	Goat	Goat anti-rabbit	TMEV
β-APP	monoclonal 22C11	Merck/Millipore MAB348	1:2000	HIER	Goat	Goat anti-mouse	axonal damage

DCX = doublecortin; Foxp3 = forkhead box protein P3; GFAP = glial fibrillary acidic protein; GrB = granzyme B; NeuN = neuronal nuclear protein; TMEV = Theiler's murine encephalomyelitis virus; β-APP = beta-amyloid precursor protein; HIER = heat induced epitope retrieval (microwave heating, citrate buffer)

Table S2. Details of primer sequences used for reverse transcription quantitative polymerase chain reaction

Gene	Acc. No.	Primer direction	Primer position in mRNA	Primer sequence from 5′→ 3′	Amplicon length
GAPDH	AY618568	Forward	1-20	GAG GCC GGT GCT GAG TAT GT	288 bp
		Reverse	288-269	GGT GGC AGT GAT GGC ATG GA	
HPRT	NM_013556.2	Forward	646-665	GGA CCT CTC GAA GTG TTG GA	188 bp
		Reverse	814-833	TCG TAT TTG CAG ATT CAA CT	
β-Actin	AY618569	Forward	1-20	GGC TAC AGC TTC ACC ACC AC	233 bp
		Reverse	214-233	ATG CCA CAG GAT TCC ATA CC	
TMEV-DA	M20301.1	Forward	1596-1615	TGG TCG ACT CTG TGG TTA CG	238 bp
		Reverse	1814-1833	GCC GGT CTT GCA AAG ATA GT	
IFN-γ	NM_008337.4	Forward	176-195	CAC GGC ACA GTC ATT GAA AG	144 bp
		Reverse	300-319	AAT CTG GCT CTG CAG GAT TT	
IL-1α	NM_010554.4	Forward	294-313	AAG CAA CGG GAA GAT TCT GA	179 bp
		Reverse	453-472	TGA CAA ACT TCT GCC TGA CG	
IL-1β	NM_008361.4	Forward	311-330	AGC TAC CTG TGT CTT TCC CG	150 bp
		Reverse	439-460	AGT GCA GTT GTC TAA TGG GAA C	
IL-4	NM_021283.2	Forward	224-245	CCT CAC AGC AAC GAA GAA CAC C	156 bp
		Reverse	358-379	CAT CGA AAA GCC CGA AAG AGT C	
IL-5	NM_010558.1	Forward	104-123	ATG GAG ATT CCC ATG AGC AC	180 bp
		Reverse	264-283	CCC ACG GAC AGT TTG ATT CT	
IL-6	NM_031168.2	Forward	250-269	GTT CTC TGG GAA ATC GTG GA	176 bp
		Reverse	404-425	CCA GAG GAA ATT TTC AAT AGG C	
IL-10	NM_010548.2	Forward	307-326	CCA AGC CTT ATC GGA AAT GA	162 bp
		Reverse	449-468	TTT TCA CAG GGG AGA AAT CG	
TGF-β1	NM_011577.2	Forward	1719-1738	TTG CTT CAG CTC CAC AGA GA	183 bp
		Reverse	1882-1901	TGG TTG TAG AGG GCA AGG AC	
TNF-α	NM_013693.3	Forward	268-287	GCC TCT TCT CAT TCC TGC TT	203 bp
		Reverse	451-470	CAC TTG GTG GTT TGC TAC GA	

Acc. No. = GenBank®-accession-number; bP = base pair; GAPDH = glyceraldehyde-3-phosphate dehydrogenase; HPRT = Hypo-xanthine-guanine phosphoribosyl transferase; TMEV-DA = Daniels strain of Theiler's murine encephalomyelitis virus; IFN-γ = interferon-gamma; IL = interleukin; TGF-β1 = transforming growth factor-β1; TNF-α = tumor necrosis factor-α.

Table S3. Details of antibodies used for flow cytometry

Antibody	Conjugate	Supplier	Clone	Dilution	Specificity
CD4	FITC	Thermo Fisher Scientific, Invitrogen, eBioscience™, Waltham, USA	RM4-5	1:100	$CD4^+$ helper T cells
CD4	PerCP Cy5.5	Thermo Fisher Scientific, Invitrogen, eBioscience™, Waltham, USA	RM4-5	1:200	$CD4^+$ helper T cells
CD8a	APC	BD Biosciences, BD Pharmingen™, Heidelberg, Germany	53-6.7	1:200	$CD8^+$ cytotoxic T cells
CD8a	PE	BD Biosciences, BD Pharmingen™, Heidelberg, Germany	53-6.7	1:200	$CD8^+$ cytotoxic T cells
CD62L	PE-Cy7	Thermo Fisher Scientific, Invitrogen, eBioscience™, Waltham, USA	MEL-14	1:200	Naïve T cells, downregulation upon T cell activation, L-selectin
CD69	APC	Thermo Fisher Scientific, Invitrogen, eBioscience™, Waltham, USA	H1.2F3	1:200	Early T cell activation, upregulation upon T cell activation
CD69	PerCP Cy5.5	Thermo Fisher Scientific, Invitrogen, eBioscience™, Waltham, USA	H1.2F3	1:200	Early T cell activation, upregulation upon T cell activation
